# Advances in the Development of Nano-Engineered Mechanically Robust Hydrogels for Minimally Invasive Treatment of Bone Defects

**DOI:** 10.3390/gels9100809

**Published:** 2023-10-10

**Authors:** Kulwinder Kaur, Ciara M. Murphy

**Affiliations:** 1 Tissue Engineering Research Group, Department of Anatomy & Regenerative Medicine, RCSI University of Medicine and Health Sciences, D02 YN77 Dublin, Ireland; kulwinderkaur@rcsi.com; 2 School of Pharmacy and Biomolecular Sciences, RCSI University of Medicine and Health Sciences, D02 YN77 Dublin, Ireland; 3 Advanced Materials and Bioengineering Research (AMBER) Centre, Trinity College Dublin (TCD), D02 PN40 Dublin, Ireland; 4 Trinity Centre for Bioengineering, Trinity College Dublin (TCD), D02 PN40 Dublin, Ireland

**Keywords:** injectable hydrogels, bone regeneration, nanoengineering, mechanically robust hydrogels, minimally invasive

## Abstract

Injectable hydrogels were discovered as attractive materials for bone tissue engineering applications given their outstanding biocompatibility, high water content, and versatile fabrication platforms into materials with different physiochemical properties. However, traditional hydrogels suffer from weak mechanical strength, limiting their use in heavy load-bearing areas. Thus, the fabrication of mechanically robust injectable hydrogels that are suitable for load-bearing environments is of great interest. Successful material design for bone tissue engineering requires an understanding of the composition and structure of the material chosen, as well as the appropriate selection of biomimetic natural or synthetic materials. This review focuses on recent advancements in materials–design considerations and approaches to prepare mechanically robust injectable hydrogels for bone tissue engineering applications. We outline the materials–design approaches through a selection of materials and fabrication methods. Finally, we discuss unmet needs and current challenges in the development of ideal materials for bone tissue regeneration and highlight emerging strategies in the field.

## 1. Introduction

The reparation and regeneration of bone tissue remain an important challenge in the field of orthopaedic and craniofacial surgery. Traumatic injuries and pathological diseases, including osteoporosis, can impair the fracture repair process, leading to non-union or delayed fractures, immobility, severe pain and deformity [1]. Such defects require clinical mediation if functional restoration and complete healing of the bone are to be achieved. The demand for bone grafts represents the second most common tissue transplantation procedure after blood. The current market in the EU for bone graft substitutes is estimated to be worth USD 4.15 billion by 2026 [2]. Currently, the gold standard of treatment for non-union and critical-sized bone defects is the use of bone grafts, which perform as a framework for new bone ingrowth. The most commonly used bone grafts are autografts, taken from the patient itself or allografts taken from an organ donor. However, both methods have confirmed and well-documented limitations including restricted donor supply and alteration of the material properties as a result of processing. The use of autografts is an invasive procedure, requiring two surgeries, one for harvesting bone and a second to treat the affected area. The consequence of these surgeries often results in further pain and complications for elderly patients and patients with pre-existing conditions. One of the most common post-operative complications following implantation is infection, which, in extreme cases can lead to loss of the affected limb [3]. Another challenge with the use of implantable bone grafts is their application in irregular or atypical-shaped defects. Grafting bone into defects with a complex geometry may result in improper defect margin adaptation, which can cause numerous complications such as improper vascularization and directional repair, leading to further pain and post-operative complications for the patient. As such, there is a clinical need for injectable bone graft substitutes that can overcome the limitations of implantable bone grafts.

### 1.1. Overview of Bone Physiology at Defect Site

It is essential to create a conducive physiochemical environment at a bone defect site to support the natural healing processes and enhance the chances of successful bone regeneration. Bone healing is a complex and dynamic process that occurs naturally in the body when a bone is broken [4]. The process involves several stages and typically takes several weeks to months, depending on the severity, location of the fracture, the individual’s age and overall health, and the treatment received. Three main stages of the bone defect healing process are inflammation, bone formation and remodelling [5].

**Figure 1:**
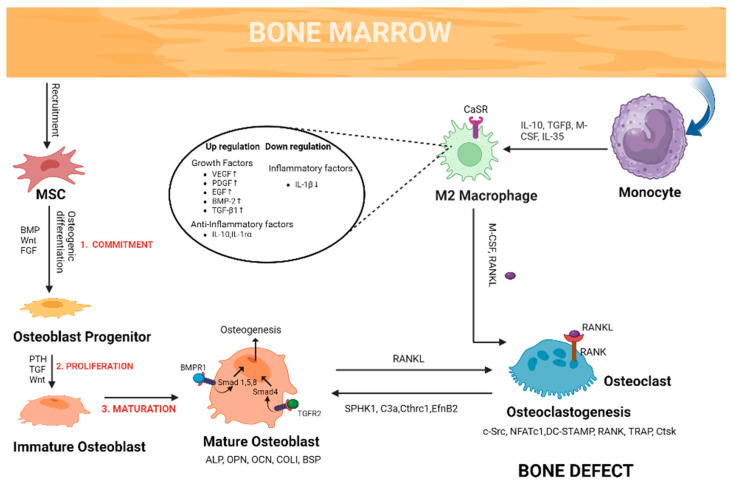
Schematic representation of different processes involved in bone healing.

When a bone is broken, the body’s natural inflammatory response is triggered. Inflammatory cells, such as cytokines, macrophages and white blood cells, infiltrate bone defect sites to clean bone-tissue debris and form vascular tissue and granulation tissue [6,7]. Macrophages under the stimulation of a hypoxic environment and cytokines polarized towards M1. They secrete a series of pro-inflammatory cytokines (IL-1, IL-6) which help to recruit mesenchymal stem cells and establish an osteogenic environment for tissue repair [4,5]. There are two repair mechanisms after that depending on the location of the bone defect: endochondral and intramembranous ossification [6]. Firstly, soft callus tissue is formed which is composed of collagen and cartilage, which helps stabilize the fracture site [8]. This soft callus is temporary but is crucial in bridging the gap between the broken bone ends. Mesenchymal stem cells along with endothelial cells and chondrocytes secrete matrix metalloproteinases to degrade cartilage matrix followed by maturation of chondrocytes into osteoblasts, which gradually replace soft callus with hard callus [9,10]. This process strengthens the fracture site (Figure 1). The final and longest stage of bone healing is bone remodelling. During remodelling, excess bone material is resorbed by osteoclasts, while osteoblasts continue to deposit new bone [11]. The crosstalk between osteoblasts and osteoclasts plays an important role in the remodelling process. The main goal of remodelling is to restore the bone’s strength, function and geometry as closely as possible to its pre-injury state [6].

### 1.2. Minimally Invasive Bone Graft Substitutes Commercially Available for Bone Repair

The most common injectable bone graft substitute used in clinics is polymethyl methacrylate (PMMA), the first generation of bone cement. It was first used by doctors as a dental material in the 1930s and was later used for femoral head replacement in 1953 and hip replacement in 1964 [4]. A number of PMMA cement-based injectable biomaterials have come onto the market, including Stryker’s Simplex^®^ (Kalamazoo, MI, USA), DePuy Synthes SMARTSET™ (Wayne, IN, USA) and Heraeus PALACOS^®^ (Hanau, Germany), ensuring PMMA remains the most commonly used injectable grafting material in orthopaedic surgery. PMMA has good plasticity and mechanical properties, widely used in the clinical treatment of spinal degeneration and osteoporotic vertebrae. However, there are certain limitations associated with PMMA, as it is non-biodegradable, non-resorbable and does not possess bioactive behaviour. Therefore, it does not facilitate bone regeneration and is not suitable for use as a bone regenerative platform. In addition, a number of clinical complications have been reported as a result of cement leakage into surrounding tissues [5]. The commercial space for injectable bone graft substitutes has expanded beyond PMMA (Table 1) to improve bioactivity. Calcium phosphate cement (CPC) was the first material approved by the Food and Drug Administration (FDA) for human use for the treatment of craniofacial defects and bone fractures. CPCs are defined as a combination of one or more calcium phosphate powders, which, upon mixing with a liquid phase, form a paste able to self-set and harden in situ in the bone defect site to form a scaffold. Since their discovery in the 1980s, there has been extensive research and development in CPC formulations. With an emphasis on mechanical properties, the majority of these products are calcium phosphate (CaP)-based bioceramics such as tricalcium phosphate (TCP) or hydroxyapatite (HA). The use of these bioceramics as bone defect substitutes for repair is inspired by the composition of bone, where these bioceramics are present in high ratios and possess biocompatibility, biodegradability, osteoconductivity and mechanical properties. Therefore, CPCs are promising for clinical application; however, despite the extensive range and longstanding use of these products, there is limited clinical data for supporting their efficacy in the context of fracture management and bone healing [6,7]. As such, there remains a need within the biomaterial research field for mechanically competent minimally invasive therapeutic biomaterials, capable of supporting load-bearing defect repair with fully functional regenerated bone tissue.

### 1.3. Biomaterial-Based Bone Graft Substitutes and Mechanical Considerations

Next-generation manufacturing and materials approaches have been used in the development of biomaterials that mimic the native bone environment and modulate the healing process, through structure and composition. Traditionally, the design and fabrication of biomaterial-based bone regenerative technologies have been based on the well-established tissue engineering triad, centred on three main components considered essential for tissue regeneration: (1) biomaterial to provide a structural and signatory platform for tissue in-growth and formation; (2) a targeted source of cells to regenerate new tissue; (3) regulatory signals to drive cell proliferation and differentiation, and ultimately tissue regeneration.

**Figure 2:**
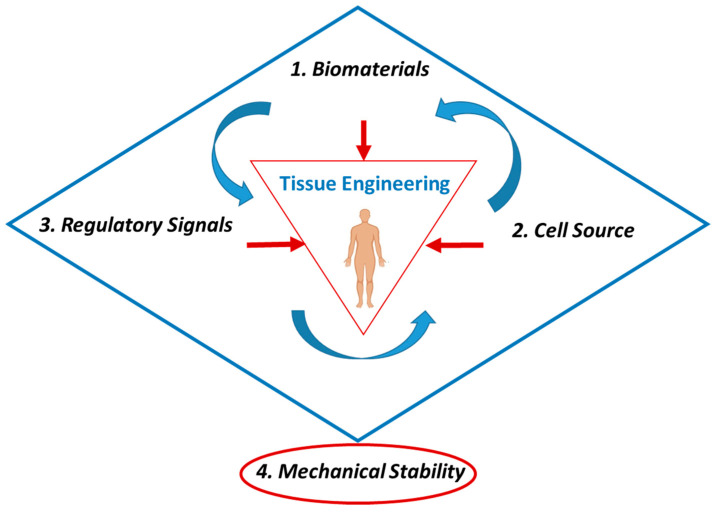
Illustration depicting the diamond concept for bone tissue engineering. The tissue triad contains the three essential components for developing tissue regenerative technology: (1) biomaterials; (2) cell source; (3) regulatory signals. A fourth component, mechanical stability, is essential for developing regenerative biomaterial platforms for bone repair.

However, the triad concept has been re-defined over the past few decades as biomaterial-based regenerative technologies have advanced. For bone regeneration and orthopaedic-centred clinical applications, a key component has been incorporated. In 2007, Giannoudis et al. introduced mechanical properties as a crucial fourth element in the development of biomaterial-based bone graft substitutes, proposing a diamond concept to replace the triad (Figure 2). Mechanical and structural functionality is essential for bone repair. From a healing perspective, it is necessary for early callus formation to bridge the fracture site and allow transmission of mechanical loading across the fracture line. The progressive maturation of the fracture callus from woven to lamellar bone is dependent on a mechanically stable fracture site. Furthermore, long bones of limbs and spinal vertebrae typically experience torsional and compressive loads during everyday activities [8,9,10] and injectable biomaterials developed for application in these bones must be able to both support the damaged architecture of the defect and withstand significant mechanical loads during the healing process. In load-bearing environments, the mechanical properties of biomaterials should be in close association with the mechanical properties of the surrounding tissue. In the case of biodegradable biomaterials, the variation in mechanical properties due to the degradation should also be compatible with the bone healing process. Advancement in biomaterial fabrication and processing techniques is driving a new era of bone graft materials with defined mechanical properties, and biological and structural properties to help bone remodelling, compared to traditional strategies. However, the number and type of commercially available biomaterial-based bone grafting materials are currently disproportionate to the volume of published scientific work on these innovative biomaterial platforms for bone healing [11]. In particular, there is a distinct lack of injectable biomaterials available for minimally invasive procedures with the requisite mechanical properties to successfully replace bone cements currently used in clinics. Developing advanced minimally invasive or injectable biomaterial strategies to safely and successfully fill complex bone defects and regenerate bone tissue in load-bearing environments is of significant clinical interest.

### 1.4. Challenges in Developing Mechanically Robust Injectable Biomaterials

Hydrogels are 3D porous networks with high water content that are formed via crosslinking among amphiphilic polymers. They have been widely researched and applied as biomaterial platforms in regenerative medicine applications, including cartilage, nerve, skin and bone, as they have a structural similarity to natural extracellular matrix (ECM), promoting cell survival, proliferation, and differentiation by providing microenvironment cues similar to ECM in terms of mechanics and architecture. Furthermore, they can be engineered for injectable applications, as hydrogels with in situ gelation properties have the ability to turn from a liquid state to a solid gel state (sol–gel transition). This characteristic is extremely favourable for the repair of atypical-shaped bone defects as the hydrogels have the potential to fill geometrically complex and irregular spaces. However, the development of injectable hydrogels for bone repair has proved extremely challenging, particularly developing injectable hydrogels that have rapid gelation rates to avoid the leakage of materials to the surrounding tissue, are biocompatible and biodegradable, with structural properties to withstand the significant and complex mechanical forces in bone [12].

Various mechanisms such as chemical, physical, catalysed crosslinking, in situ double network formation and the incorporation of particulate reinforcing agents, such as nano-particles/nano-fillers, have been applied in the development and structural enhancement of injectable hydrogels for application in load-bearing environments [13]. However, increasing stiffness through crosslinking and the incorporation of nano-fillers has been shown to significantly alter the shear-thinning potential of the hydrogels, making it complicated to deliver them in an injectable manner. This drawback confines the utilization of hydrogels in clinics and has motivated researchers to design new hydrogels that can be delivered in an injectable manner. Specifically, mechanically robust injectable hydrogels require the hydrogel solution to be stable in the pre-injection state but provide the required mechanical reinforcement after injection without additional mechanical inputs.

Degradation and resorption of hydrogels is also an important consideration in mechanically robust hydrogel design parameters. Time-dependent degradation is desirable in many applications whereby the degradation rate of hydrogel should match the rate of tissue regeneration. In addition, the by-products of degradation must be non-toxic. Many hydrogels degrade rapidly, especially in electrolyte solutions, with degradation often being accompanied by the generation of acidic by-products [14,15]. These restrictions have drawn attention to designing hydrogels with enhanced toughness and stretchable physiochemical properties. However, fabricating mechanically robust injectable hydrogels that are chemically and morphologically stable for clinical use remains a challenge. For example, tailoring the material characteristics by the adjustment of polymer content and the crosslinking density can limit the nutrient and ions transport throughout the matrix in physiological conditions [16,17,18], which can lead to unwanted swelling and reduction in mechanical properties. Indeed, many mechanically robust hydrogels reported to date cannot maintain their mechanical properties during tissue culture or after implantation [19,20,21]. Thus, there remains a need to develop high-water-content, mechanically robust injectable hydrogels that can extend the lifetime of clinically suitable materials by dependably avoiding degradation under aggressive in vivo conditions.

This review presents current advances and available tools in the field of nano-engineered mechanically robust injectable hydrogels for minimally invasive bone regenerative applications while highlighting barriers in translating such materials for use in the clinic. We begin by introducing commonly employed strategies to mechanically strengthen injectable hydrogels and go on to discuss the promising advances in nanotechnology that are paving the way for mechanically robust injectable hydrogels for bone regenerative applications. While many of the technologies discussed are not yet feasible for clinical applications, this review aims to present the promising in vitro and in vivo data available in the literature to highlight the potential use of nano-engineered mechanically robust hydrogels to tackle the challenging and pressing clinical orthopaedic needs.

## 2. Design and Materials Considerations for Mechanically Robust Injectable Hydrogels

**Figure 3:**
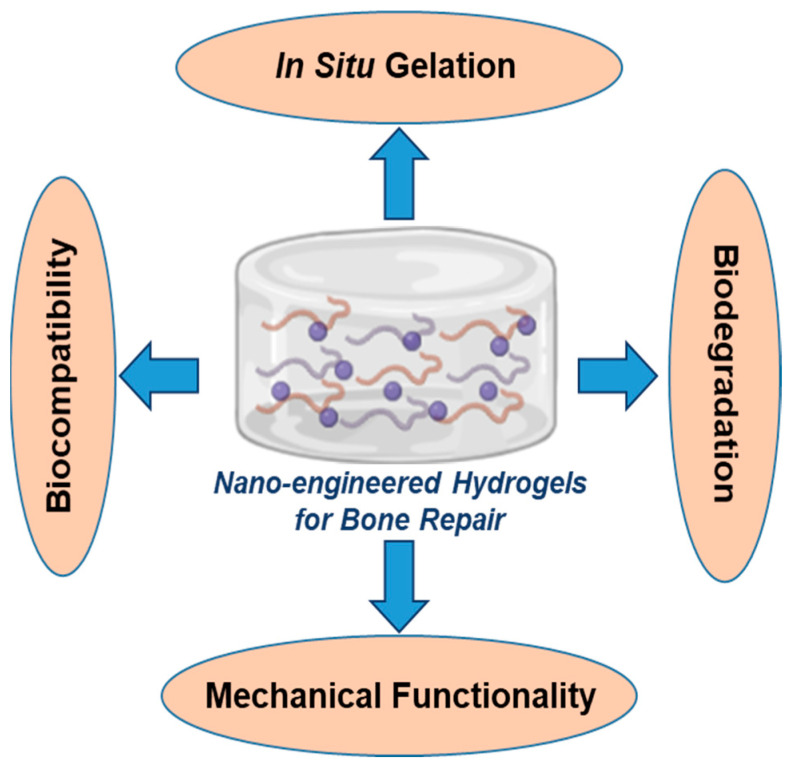
Key hydrogel properties required for bone repair applications.

To design mechanically robust injectable hydrogels with precisely tuned properties, assessing the clinical requirements, experiment designs and procedures are critical to determine which basic factors lead to material failure in conventional hydrogels. In the design of nano-engineered injectable hydrogels, the most important considerations are delivery and retention within a specific site, mechanical properties comparable to native bone, controlled degradation and biocompatibility (Figure 3).

### 2.1. In Situ Gelation and Biocompatibility

Upon injection, it is essential that injectable hydrogels solidify rapidly to avoid unnecessary diffusion into the surrounding tissues. Furthermore, the materials used and by-products released must be biocompatible to minimize cell toxicity. Injectable hydrogels, in rheological terms, behave as a fluid while injecting (i.e., elastic modulus (G′) < storage modulus (G″)), and turn into a solid (G′ > G″) post injection, to retain the hydrogel within the desired region. Crosslinking between functional groups of different polymers or components in hydrogels is an important and responsible factor for thermally driven sol–gel transition. The addition of crosslinking agents between the polymer chains to produce the hydrogel affects the sol–gel transition of the hydrogels, depending upon the type and degree of crosslinking. In situ gelation of the hydrogels can be obtained by either physical or chemical crosslinking through polymerization of monomers, or through covalent bonding in between polymer chains. Crosslinking modifies the microstructure, mechanical properties and biocompatibility of the hydrogels. The degree of modification is determined by the extent and type of crosslinking used. Physical crosslinking creates hydrophobic interactions within polymeric chains and is generally responsible for thermally driven gelation of hydrogels. Chemical crosslinking occurs through chemical moieties. Some of the most important and rapid reactions are click chemistry, Schiff base, Michael addition and enzyme-catalysed reactions. Alternatively, sol–gel transition of hydrogels can be initiated by UV light polymerization of polymeric chains, in the presence of free-radical groups and a photo initiator. An ideal crosslinking reaction must produce hydrogels that exhibit good structural properties with minimal toxic reaction byproducts. Additionally, it is essential to consider whether the crosslinking techniques used are suitable for the required in vivo application, specifically the retention and stability of the material post injection. For example, mixing-induced two-component hydrogels (MITCH) formed through transient, non-covalent crosslinking, produce hydrogels with shear-thinning properties ideal for minimally invasive applications. However, these hydrogels lack long-term stability post injection due to the non-covalent crosslinks formed [22], rendering this crosslinking approach unsuitable for long-term hydrogel applications, particularly in bone. In 2018, Lou et al. used a biocompatible benzimidazole-based organocatalyst to chemically crosslink hyaluronic acid (HA), producing hydrogels for encapsulated cell delivery, with temporally modulated high stability. The hydrogels produced demonstrated enhanced injectability and long-term stability tailored for cell delivery at various time-points of application [23]. In 2006, Zanello et al. reported the use of carbon nanotubes (CNTs) for osteoblast growth and bone formation. They concluded that CNTs having neutral electric charge leads to the highest rate of cell growth [24]. In 2015, Cai et al. developed an injectable double network hydrogel, which goes through two different physical crosslinking procedures, to deliver human adipose-derived stem cells. The first crosslinking involves ex vivo encapsulation of cells through peptide-based molecules into weak polyethylene glycol (PEG)–poly N-isopropyl acrylamide homopolymer (PNIPAm-P)-based hydrogel that dissipates force while injecting. The second step of crosslinking facilitates the in situ formation of a reinforced polymeric network that significantly delays the biodegradation of material and extends cell encapsulation time compared to single network hydrogels [25].

### 2.2. Degradation and Mechanical Properties

Hydrogels provide support as an extracellular matrix (ECM) framework for the proliferation and differentiation of cells, and tissue formation. Furthermore, they can be tailored to have biodegradable profiles that facilitate and match the ingrowth of newly regenerated tissue, such that the gradual decrease in structural support provided by the degrading hydrogels is compensated by the gradual increase in mechanical support provided by the new tissue [26]. It is essential that the by-products released during the degradation process do not interfere with the process of cell differentiation and tissue formation. For example, the biodegradation of hydrogels developed for bone regeneration should not significantly alter the local pH, which could weaken the mineralization process [27,28,29]. Most hydrogels are designed to degrade with water diffusion; however, there are materials that break down in the presence of external stimuli including pH, temperature, redox reactions and enzymatic activity [29]. Achieving optimal degradation profiles that facilitate the ingrowth of new tissue is a major challenge in developing hydrogels suitable for minimally invasive bone applications.

Biomaterial-based bone graft substitutes require mechanical functionality to support the diseased or damaged bone during the healing process. While the specific mechanical properties will vary depending on the site and application of the bone graft substitute, they are generally required to bear local compressive loads to prevent the collapse of the growing new bone tissue [30,31]. For example, biomaterials developed for vertebral bone applications must have mechanical properties suitable to withstand at least 200 N load experienced in the lumbar vertebrae while standing [32]. Injectable hydrogels, due to their high water content and soft structural properties, have inadequate mechanical properties for bone applications, which has significantly hindered their translation to the commercial and clinical arena.

To date, increased mechanical properties in hydrogels can be achieved by increasing the degree of crosslinking or reinforcing the polymeric matrix with nanofillers. Physical crosslinking interactions between polymeric chains and nanofillers, or in between nanofillers, have been exploited to form composite hydrogels or improve the mechanical properties of existing hydrogels [33,34,35,36,37]. In 2018, Wang et al. studied the effect of photoactive bis (acyl) phosphane functionalized cellulose nanocrystals (CNCs) with mono-functional methacrylate on improving elastic modulus and shape persistence of free-standing 3D structures. They reported that by increasing the content of functionalized CNCs from 3.27 to 6.14 wt%, elastic modulus increased from 2.5 to 5.5 kPa [38]. In 2011, Gaharwar et al. demonstrated the effect of combining PEG with nano-hydroxyapatite in photo-crosslinked hydrogels and reported an increase in Young’s modulus from 3.7 kPa to 15.1 kPa with the addition of nano-hydroxyapatite from 0 to 15 wt% in hydrogel network [39]. Eslahi et al. (2016) showed a 6-fold increase in storage modulus with the addition of nanoclay in their hydrogels. Nanoclay leads to the formation of physical crosslinks between the polymer matrix and nanoclay, which greatly enhances the mechanical properties [40]. Demirtaş et al. (2017) reported the improvement of elastic modulus with incorporation of nano-hydroxyapatite in 3 *w*/*v*% alginate matrix from 3.5 to 18.8 kPa and in 2 *w*/*v*% chitosan from 4.6 to 15.0 kPa [41]. Another interesting example is the use of bi-functional silica nanoparticles (NPs) that are able to crosslink via amine groups and conventional covalent crosslinking through acrylate groups in the hydrogel network. In 2020, Sujan et al. showed a significant increase in mechanical properties of poly (acrylic) acid hydrogels, whereby their tensile strength (275 kPa) was found to be ~8 times greater than conventionally crosslinked hydrogels through N,N′-methylene bisacrylamide (MBA) that have a tensile strength of 43 kPa. It was also reported that the temporary crosslinked hydrogels have high swelling capacity due to the breakdown of temporary crosslinking on immersing in aqueous media, leading to the absorption of large amounts of water without degrading the hydrogels [42]. In 2012, Shin et al. found that reinforcement of carbon nanotubes (CNTs) in GelMA resulted in a remarkable increase in the compressive modulus of ~300% and that of tensile modulus to ~400% without affecting cellular ingrowth driven by the formation of well-organized nanofibre network inside the hydrogel [43]. Our research group incorporated carboxylic acid functionalized single wall carbon nanotubes (COOH-SWCNTs) into chitosan–collagen hydrogel matrices to successfully formulate mechanically robust injectable hydrogels. Incorporation of COOH-SWCNTs increased the crystallinity of the hydrogels, leading to aligned structure and ultimately increasing the Young’s modulus of the hydrogels by 63%, up to ~4 MPa, which is coming close to that of trabecular bone [44].

While these studies are examples of the different types of nano-agents that can be used to reinforce hydrogel matrices to enhance mechanical properties, other studies have shown the importance of balancing both the interactions between the nanoscale reinforcing agents themselves, as well as with hydrogel matrix to achieve the required stiffening effect. In 2012, Liu et al. synthesized polyacrylamide (PAM)/graphene oxide (GO) nanocomposite hydrogels with GO nanosheets as crosslinkers [45] and investigated the mechanical properties of these hydrogels compared to conventional PAM hydrogels crosslinked chemically with N,N′-methylenebisacrylamide. While the mechanical properties of the hydrogels were measured via the type and content of crosslinkers, the authors demonstrated a 4.5-fold increase in tensile strength in the hydrogels crosslinked with GO nanosheets. However, in 2021, Ligorio et al. prepared physically crosslinked self-assembled peptide hydrogels reinforced with GO sheets. They reported a storage modulus of ~1.7 kPa with 0.5 wt% GO, a 2-fold increase over peptide-only hydrogels without GO [46] due to hydrophobic interactions between GO and the self-assembled peptides.

## 3. Approaches to Fabricate Nano-Engineered Mechanically Robust Injectable Hydrogels

**Figure 4:**
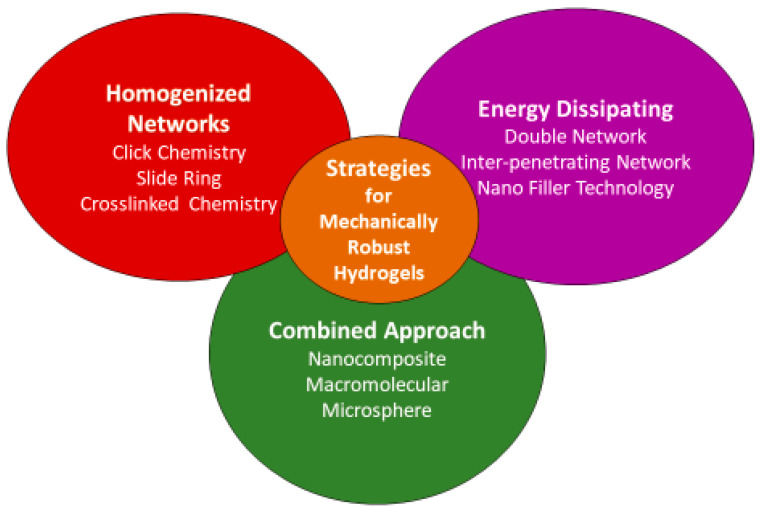
Strategies for preparing mechanically robust hydrogels.

The formulation and fabrication process can significantly influence the physicochemical properties and structure of hydrogels. As such, there is a large volume of research published on the use of different materials and crosslinking techniques to achieve improved mechanical properties in hydrogels, which are traditionally weak. They are summarized in Figure 4 given below.

(1) Homogeneous hydrogels formulated using chemical crosslinking including click chemistry, Michael additions, dynamic covalent bonding and enzymatic crosslinking;

(2) Spontaneously formed hydrogels using physical crosslinking, specifically temperature, pH-sensitive hydrogels, interpenetrating network (IPN), double network (DN) and fibre-reinforced hydrogels;

(3) Hydrogels formulated using multifunctional crosslinkers.

### 3.1. Homogeneous Hydrogels Formulated Using Chemical Crosslinking

Conventional hydrogels usually have heterogeneous polymeric networks due to poorly controllable crosslinking methods. If hydrogels are synthesized under controlled conditions and environment, it will improve network connectivity and the resulting hydrogels would have improved mechanical properties with evenly distributed load over polymeric network chains. Here, we do not aim to give a full summary of mechanically robust hydrogels, but introduce mechanically robust and biodegradable hydrogels that have had attention recently. The section given below discusses some approaches typically employed to synthesize homogeneous hydrogels with superior mechanical properties for application in the repairing of bone defects.

#### 3.1.1. Click Chemistry

A protocol to synthesize hydrogels with enhanced mechanical properties via controlled design is known as “click chemistry”. It refers to a synthetic concept involving reactions that are rapid in kinetics, proceeded through the connection of small units, high yielding, high selectivity, wide in scope, stereospecific and generate non-toxic by-products that are less reactive towards cellular components [47]. Reactions involving click chemistry include nucleophilic ring opening, non-alkyl carbonyl [48,49], Diels–Alder [50], copper-catalysed azide-alkyne cyclo-addition [51,52], tetrazine–norbornene chemistry [53], thiol-epoxy [54], carbon–carbon multi bond addition, thiol–ene [55,56] and thiol–maleimide couplings [57]. These reactions usually need a catalyst or initiator but the use of these can inhibit the bioactive potential of hydrogels [48]. In 2019, Hu et al. prepared mechanically robust injectable hydrogels by using hydroxyapatite and pH-sensitive bi-functional acetylated β-cyclodextrin NPs through Diels–Alder click chemistry and dynamic covalent. The storage modulus of the developed hydrogel was 3000 Pa with the addition of the acetylated β-cyclodextrin NPs [58]. In 2017, Buwalda et al. reported copper (I)-catalysed cycloaddition reaction, diacetylene functionalized and tetra-azide-functionalized PEG derivatives were used to form a mechanically robust PEG-based hydrogel with an organized network structure [59].

In 2016, Kaga et al. developed an injectable hydrogel based on dendron–polymer–dendron conjugates via radical thiol–ene “click” reaction. In this reaction, the dendron–polymer conjugates were prepared via an azide-alkyne “click” reaction of alkene-containing polyester dendron, having an alkyne group at their endpoint, with linear PEG-bisazides. The sequential thiol–ene “click” reaction uses a tetra thiol-based crosslinker to crosslink these alkene-functionalized dendron–polymer conjugates, thus resulting in clear and transparent hydrogels [60]. However, despite controlled crosslinking and rapid gelation times achieved using thiol–ene and thiol–yne click chemistry reactions, potential toxicity from photoinitiators and radicals, along with cross-reactivity with thiols, remains concerning in the resultant hydrogel [60]. Therefore, it is important to develop initiator and catalyst-free reaction systems for the preparation of biocompatible injectable hydrogels [61,62] as reported by Hunag and Jiang [63]. They developed the catalyst-free injectable hydrogel by varying the concentration of carboxymethyl chitosan (CMC) and using amino–yne click chemistry. The benefit of click chemistry for developing injectable hydrogels is the likelihood of tailoring the properties of the materials for highly crosslinked regimes to get full-interpenetrated hydrogels, allowing for chemical modification to result in a diverse collection of mechanically robust injectable hydrogels. Overall, complex synthesis routes and impending side reactions between biomolecules and the hydrogels should be considered when selecting click chemistry protocols for preparing nano-engineered mechanically robust injectable hydrogels for biomedical applications.

#### 3.1.2. Michael Addition

The Michael addition is one of the in situ reactions that involves the conjugate addition reactions of a nucleophilic negative carbon ion (electron donor) with an electrophilic conjugated ion (electron acceptor) and vice versa. It is a commonly used method to fabricate injectable hydrogels, due to high selectivity under ambient conditions and controllable reaction time [64,65,66,67,68]. In 2020, Zhu et al. developed nano-engineered injectable shear-thinning hydrogels by using the Michael addition. They used nanosized cationic micelles of methoxyl PEG-block-poly (ε-caprolactone) and poly (ε-caprolactone)-block-poly (hexamethylene guanidine) hydrochloride-block-poly (ε-caprolactone) and added sodium carboxymethyl cellulose into the micellar solution, resulting in a homogenous shear-thinning electrostatic hydrogel for medical applications [69]. In 2020, Rajabi et al. developed a nano-engineered mechanically robust injectable hydrogel for tissue engineering applications by using gelatin methacrylate and thiolated gelatin with polydopamine functionalized Laponite^®^. This hydrogel was fabricated via the Michael addition in between gelatin methacrylate and thiolated gelatin, and covalent crosslinking with polydopamine functionalized Laponite^®^ which improved mechanical properties (tensile strength 22–84 kPa and compressive strength 54–153 kPa) of the resulted hydrogel [70]. In 2015 and 2016, Rodell et al. used the same host–guest chemistry with Michael addition crosslinking to prepare mechanically robust HA injectable hydrogels with a compressive modulus of ~230 kPa by altering the concentration of the host–guest network and the ratio of methacrylate: thiol groups. They found a ~100-fold increase in compressive modulus as compared to the modulus accomplished with single network gel [71,72].

#### 3.1.3. Dynamic Covalent Bonding

Dynamic covalent bonding was used for developing nano-engineered mechanically robust injectable hydrogels which include Schiff base, hydrozone, borate and oxime reactions [73]. These covalent bonding can take place under ambient conditions or can be initiated by pH [74], temperature [75] and redox reactions [76]. The chemical stability of the bonding helps the solution to gelation transition and degradability of the developed hydrogels [77]. Schiff base is a conventional dynamic covalent bond also known as an imine bond. They were generated from aldehyde and amine functional groups without using any external or internal stimuli under ambient conditions with high reaction rates [78,79,80]. In 2019, Zhang et al. developed nano-engineered mechanically robust injectable hydrogel by using aldehyde-functionalized cellulose nanocrystals and collagen through dynamic Schiff base bonds for biomedical applications. They reported that the hydrogels possessed improved injectability and elastic properties with the addition of nanocrystals [81].

In 2020, Panita et al. reported nano-engineered injectable hydrogels with comparable physiochemical properties and mechanical strength to native bone, which also promoted bone regeneration. They used cellulose nanofibres/nanocrystals and collagen for preparing hydrogel via covalent crosslinking by a Schiff base reaction. They found that the addition of cellulose nanofibres/nanocrystals significantly improved the mechanical properties from ∼28 kPa to ∼379 kPa and gelation time reduced from 24 s to 7 s [82]. In 2018, Ren et al. used a different approach to enhance the mechanical properties of Schiff base hydrogels by adding a microsphere of hydroxyapatite and calcium carbonate to oxidized alginate and carboxymethyl chitosan hydrogel for bone tissue engineering. They reported a sharp increase in compressive strength from 64.2 ± 5.7 kPa to 276.8 ± 18.9 kPa [83].

Unlike Schiff base, the reaction rates of hydrazine and oxime are slow at neutral pH but form covalent bonds which are stronger due to the steric hindrance effect [84]. In 2015, Domingues et al. developed a nano-engineered mechanically robust injectable hydrogel formed by hydrazone crosslinking. They mixed hydrazide-functionalized HA with aldehyde-modified cellulose nanocrystals and found a 2.7-fold increment in compressive modulus with the addition of nanocrystals at 0.25 wt% content [85]. In 2015, Hardy et al. developed injectable hydrogel by using linear aminooxy-terminated PEGs and aldehyde-modified HA. A reaction between these two resulted in oxime crosslinking in the injectable hydrogel. This hydrogel can load collagen-I and human marrow stromal stem cells for potential use in bone tissue engineering as an injectable matrix [86]. Borate-based hydrogels are important intelligent materials and were studied numerously for developing glucose-sensitive materials [87] and injectable hydrogels [88,89].

In 2017, Pettignano et al. developed injectable hydrogels at two different pH values (neutral or basic) of alginate, modified by boronic acid. The mechanism of gelation was through the formation of boronate ester bonds between the vicinal diols and boronic groups on the pyranose rings [90]. In 2018, Zhao et al. developed harder and mechanically robust injectable hydrogels by using poly (vinyl alcohol) (PVA) as the pillar to react with 4-carboxyphenylboronic acid (CPBA) for borate linkage, and Ca^2+^ a multivalent ion contributed to further electrostatic crosslinking with CPBA. The developed hydrogels had compressive modulus ~1 MPa [91]. In 2011, Choh et al. reported a controlled way for preparing hydrogels by exchange reaction between PEG-dithiol and pyridyl-disulfide modified HA which forms disulfide crosslinking between PEG and HA [92]. In 2017, Yu et al. fabricated injectable hydrogels in the pH range from mildly acidic to basic values. They used a cyclic disulfide group onto polyethylene oxide (PEO) for crosslinking the thiol functionalized F127. In general, disulfide bond-containing hydrogels are responsive to reductive agents, as Schiff bases are responsive to acids, and borate bonds respond to glucose for generating injectable hydrogels [93].

#### 3.1.4. Enzyme-Mediated Crosslinking

**Figure 5:**
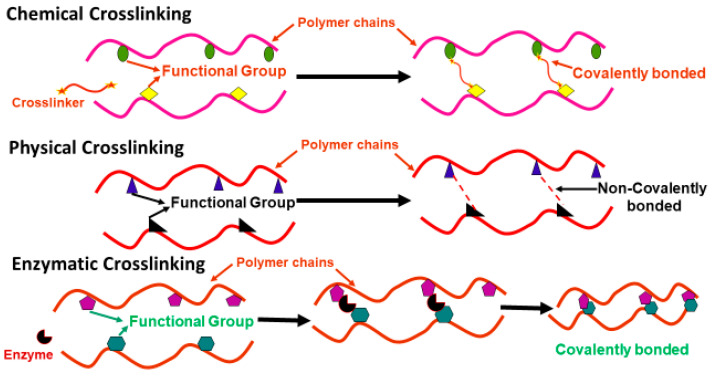
Schematic of different crosslinking approaches in hydrogels.

The use of enzymes as crosslinkers is another process useful for the preparation of novel injectable hydrogels (Figure 5). All natural biomacromolecules, such as proteins and nucleic acids, are produced in living organisms via enzymatic catalysis. Enzymes are biocatalysts and catalyse all metabolic reactions in vivo to maintain life [94,95]. Enzymatic crosslinking has been used numerously in hydrogel preparation due to the fast gelation at ambient conditions, low cytotoxicity, high site specificity and enhanced mechanical properties [96,97,98]. According to the Enzyme Commission, enzymes are categorized into main six groups, i.e., transferases oxidoreductases, lyases, hydrolases, ligases and isomerases. Recently, the oxidoreductases, transferases, and hydrolases enzyme groups were used to form the covalent crosslinks in gelling polymeric matrices for BTE applications [98,99]. Enzymatic crosslinking is a safer mechanism than other chemical crosslinking methods discussed above; however, it is restricted by the type of reactions and enzymes for wider applications [100].

In 2019, Bi et al. prepared injectable hydrogels of tyramine-modified carboxymethyl chitin (CMCH-Tyr) through enzymatic crosslinking and found that resultant hydrogels have significantly higher compressive modulus [101] than physically crosslinked CMCH [102,103]. In 2015, Bocharova et al. developed alginate-based nanogel with tunable size using laccase both as a template and catalyst. Laccase crosslinked nanogel layers were formed in situ by a laccase-induced oxidation of iron (II) to iron (III) along with alginate chelation and deposition to form the nanogel layers [104]. In 2013, Su et al. developed nano-engineered mechanically robust hydrogels by using DMAA as the monomer, acryloylated human serum albumin as the crosslinker, and by using silica NPs as strengthening agents. The transparent hydrogels were produced after ~6 min of polymerization with compressive strength as high as 2000 kPa [105]. In 2015, Liao et al. developed nano-engineered mechanically robust hydrogel by using negatively charged calcium niobate nanosheets (CNOs) as the crosslinker. Positively charged horseradish peroxidase (HRP) was added to negatively charged CNO solution, which self-assembled onto the CNO surface via electrostatic interactions. With the glucose oxidase (GOx) and acetylacetonate (ACAC), the layered CNOs complex was in situ scale off by the polymerization of poly (ethylene glycol) methacrylate (PEGMA) to additionally crosslink the macromolecules through supramolecular interactions [106].

### 3.2. Spontaneously Formed Hydrogels Using Physical Crosslinking

An alternative approach to developing nano-engineered mechanically robust injectable hydrogels for bone tissue regeneration focuses on manipulating the concentration of weak noncovalent bonds that respond to the changes in surrounding pH and temperature values [107,108,109,110].

#### 3.2.1. Temperature-Responsive Injectable Hydrogels

Injectable hydrogels that are responsive to temperature have great potential in the field of minimally invasive technology. These sensitive hydrogels are attractive for use in BTE applications due to their ability to undergo sol-to-gel transition at ambient temperature in situ [111,112]. Chitosan [113], collagen [114], poly(lactic co-glycolic acid)–PEG [111], poly(ethylene glycol-b-[DL-lactic acid co-glycolic acid]-b-ethylene glycol) [115] and PNIPAm [112,116] are widely used polymers for temperature-sensitive hydrogel because of their lower critical solution temperature (LCST) and ability to create hydrogen bonding (inter and intramolecular), which is based on changes from sol-to-gel state for applications in bone tissue engineering [117]. Such polymeric systems were further decorated with appropriate NPs to develop mechanically robust injectable temperature-responsive hydrogels.

In 2016, Zahra et al. developed a nano-engineered mechanically robust thermoplastic starch/ethylene vinyl alcohol polymer network for BTE. Nano-structured forsterite was used as the strengthening agent in the polymeric matrix for improving mechanical and biological properties. Mechanical properties of the composites containing 5 and 10 wt% nanoforsterite fall were in the range of the cancellous bone. A significant increase in proliferation of human osteoblast MG63 cells was observed in hydrogels with forsterite compared to controls [118]. In 2016, Moreira et al. prepared mechanically robust nano-engineered thermoresponsive injectable hydrogels by using chitosan and collagen reinforced with bioactive glass NPs. They found that sol-to-gel transition occurred at 37 °C [119]. In 2009, Couto et al. developed thermoresponsive nano-engineered injectable hydrogels by using chitosan/β-glycerophosphate reinforced with bioactive glass NPs for orthopaedic applications [120].

#### 3.2.2. pH-Responsive Injectable Hydrogels

Hydrogels sensitive to pH show considerable potential in various biomedical applications. For developing pH-responsive injectable hydrogels, the addition of a pH-responsive molecule such as the polyelectrolyte olyacrylic acid [121], N-palmitoyl chitosan [122], oligomeric and sulfamethazine oligomers (SMOs) [123], sulfamethazine [124] is mandatory. Kim et al. [109] developed a pH-responsive injectable hydrogel with the addition of pH-sensitive SMOs at both ends of a temperature-responsive poly (ε-caprolactone-co-lactide)–PEG–poly (ε-caprolactone-co-lactide) (PCLA–PEG–PCLA) block copolymer for BTE. They demonstrated the pH-responsive SMO–PCLA–PEG–PCLA–MO injectable hydrogels underwent sol-to-gel transition at ambient temperature conditions and ~pH 7.4, and had the potential to facilitate mesenchymal stem cell differentiation in vivo.

In 2020, Panita et al. prepared pH/thermoresponsive nano-engineered injectable hydrogel with mechanical properties equivalent to bone microenvironment. They used chitosan and cellulose nanofibres/nanocrystals (CNFs/CNCs)) for preparing the injectable hydrogel. CNFs/CNCs were used as reinforcing agents for enhancing the mechanical properties of the chitosan gel to mimic the native bone tissue (from ~28 kPa to ~379 kPa) [82]. In 2009, Chiu et al. developed pH-responsive injectable hydrogels by using N-palmitoyl chitosan (NPCS), which goes through a quick nanostructure transformation because of hydrophobic interactions within a narrow pH range of (~6.5–7.0) [82].

#### 3.2.3. Interpenetrating Polymer Networks Based Injectable Hydrogels

Hydrogels formed by mixing two or more polymer chains crosslinked via noncovalent bonds are known as ‘interpenetrating polymer network hydrogels (IPNs)’. IPN hydrogels are mechanically robust due to the physical crosslinking of two polymer networks with different physical and chemical properties. These hydrogels are designed in such a manner that the ductile network of the hydrogel acts to sustain large deformation while the brittle network of the hydrogel dissipates stress throughout the hydrogel network [125]. There are two fabrication methods used to fabricate IPNs: (1) Simultaneous IPNs, which are fabricated by simultaneously polymerizing one network by a condensation reaction, while the other network is formed by a free radical reaction. (2) Sequential IPNs, whereby a single hydrophilic polymer network is crosslinked with a second polymer network to form the resulting hydrogel [126,127,128].

The advantage of IPNs is stiffness; hydrogel matrices produced have better stiffness and controllable mechanical properties as compared to single-component hydrogels in a wide range [126]. In 2013, Glassman et al. developed a physically crosslinked mechanically robust injectable hydrogel by using self-assembled peptide domains as reinforcing agents with thermoresponsive PNIPAm-based polymers. They were crosslinked via self-assembly of the peptide domains and thermogelation of NIPAM blocks, resulting in gels with a high storage modulus of ~60 kPa at physiological conditions, which was 2–3 fold stiffer than single crosslinked hydrogels containing only self-assembled peptide or NIPAM blocks [129].

In 2021, Bai et al. prepared self-reinforcing mechanically robust hydrogels by combining polyacrylamide functionalized with adamantane/β-CD host–guest supramolecular groups and PEG/chondroitin sulfate crosslinked through Diels–Alder crosslinking for bone repair. The double crosslinking approach in the hydrogels resulted in a compressive modulus of 30 MPa, which was ~15-fold higher than hydrogels prepared without the host–guest supramolecular groups [130]. Truong et al., in 2015 developed chemically crosslinked hydrogels by using fully orthogonal thiol–yne and Diels–Alder. The compressive modulus of the prepared hydrogel was 15.5 MPa, which was 6 and 120-fold higher than the dense thiol–yne and Diels–Alder single-networks, respectively [131]. On increasing the thiol content from 8 to 32 mol%, they found that the shear modulus increased from 600 to 2200 kPa. However, further increasing the thiol content decreases the shear modulus due to the interruption of the Schiff base interactions by enhanced disulfide formation. This is a major issue when two types of crosslinking occur based on a single precursor polymer [132].

In 2017, Azevedo et al. prepared a chemically crosslinked gel based on catechol-functionalized chitosan, unmodified chitosan and genipin. In these hydrogels, they found that both the catechol groups and genipin can form covalent bonds with free chitosan amine groups [133]. Subsequently, iron (Fe^3+^) complexation adds an additional physical crosslink into the system to achieve a compressive modulus of ~49.6 kPa, higher than single component networks prepared with only catechol and genipin crosslinking (~16.6 kPa), only Fe^3+^ crosslinking (~1.6 kPa), or double network hydrogels without Fe^3+^ complexation (~28.5 kPa) [133]. In 2018, Wang et al. utilized aldehyde–hydrazide crosslinking joined with phenyl boronic acid–catechol boronate ester crosslinking to form double chemical crosslinking IPN hydrogels with a pH-responsive shear modulus of ~5 kPa at pH 7 and ~1 kPa at pH 10 or pH 3 according to the relative ionization profiles of the phenyl boronic acid and catechol moieties [100]. In 2018, Zhao et al. developed chemically crosslinked poly (vinyl alcohol) (PVA) hydrogels with 4-carboxyphenylboronic acid (CPBA). CPBA acts as a crosslinker for the dual crosslinking of hydrogels through covalent and ionic bonding [91]. Furthermore, they use calcium chloride (4 wt%) to provide a two-fold increase in storage modulus (~30 kPa) with 7 wt% PVA and 3 wt% CPBA compared to those prepared without calcium chloride [91].

In 2017, Yan et al. developed covalent/ionic crosslinked orthogonal networks of chemically crosslinked PEG-chitosan Schiff base networks with an ionically crosslinked calcium–alginate network. Modulus was increased by ~7.5-fold (~15 kPa) in comparison to the single component network of the same composition [134]. In 2018, Kevin et al. developed POEGMA networks by copolymerizing cationic and anionic co-monomer in the pre-polymers. This leads to a 3-fold increase in shear storage modulus as compared to uncharged aldehyde-functionalized pre-polymers prepared via gelation using the same concentration [135].

Founded by Gong et al. in 2010, double network (DN) hydrogels are a special class of IPN hydrogels. DN hydrogels are usually fabricated by mixing two polymer networks. The primary network is stiff yet brittle and the second is ductile [18]. Ideally, DN hydrogels have water content as high as 90% and possess hardness, strength and toughness with elastic modulus in the high MPa range [136,137,138,139]. Important features of DN include the following: (1) A rigid polyelectrolyte primary network with a secondary ductile neutral polymer network. (2) A large molar ratio of the second network to the first network. (3) The first network will be tightly crosslinked, while the second will be slightly crosslinked or not crosslinked. (4) The molecular weight of the second polymer should be very high [18,136,140].

Conventionally, DN hydrogels are fabricated through the same steps as IPNs, i.e., two-step synthesis via free radical and condensation polymerization processes, one-pot synthesis, multistep stent and free shapeable method [140,141,142,143]. Biopolymer-based DN hydrogels, bi-layered mechanically robust, ultrathin, void, micro gel-reinforced particle, self-assembled and liquid crystalline DN hydrogels are some of the novel methods, which improve the mechanical strength of DN hydrogels compared to conventional physically/chemically crosslinked hydrogels [144,145,146,147,148,149,150,151]. In 2015, Tsukeshiba et al. prepared DN hydrogels by using a highly crosslinked first network of poly (2-acrylamido-2-methylpropanesulfonic acid) (PAMPS) and linear polyacrylamide (PAAm) as a second network void DN hydrogel method. They found that the mechanical properties of the DN hydrogels dramatically increased when chains of the second network (PAAm) twisted tightly with each other. This twisting in between the PAAm network plays a key role in improving the mechanical properties of DN hydrogels [138]. However, additional investigation is required to design and fabricate DN hydrogels with multifunctional properties such as biodegradability, biocompatibility, and osteoconductivity suitable for BTE.

### 3.3. Hydrogels Formulated with Multifunctional Crosslinkers

To develop hydrophilic injectable mechanically robust hydrogels, the use of nanofiller, which can act as part of hydrogels is an alternative solution to manipulate the properties of the hydrogel, i.e., mechanical and structural properties. The properties of the resultant hydrogel are the sum of the properties of the nanofiller and the hydrogel polymeric matrix. Nanoparticle-based injectable hydrogels are a type of crosslinked polymer network inflated with NPs that provide the hydrogel with better mechanical properties. These NPs can act as crosslinkers to crosslink the hydrogel, can be absorbed into polymeric chains, or can be a physical trap within the polymer network to manipulate the properties of the hydrogel [152]. The multifunctional crosslinking through NPs allows better control over crosslinking volume, density and distance between inter-crosslinking chains, which leads to better load distribution within hydrogel networks and better mechanical properties as given in Table 2. Furthermore, the use of nanofillers has been shown to confer improved bioactivity and biocompatibility to the hydrogels, beneficial for BTE [44].

In 2002, Haraguchi and Takehisa were the first to report on the use of an inorganic clay as a nanofiller to manipulate the structural properties of a PNIPAm-based nanocomposite hydrogel [153]. These fillers could be nano or micro in structure and covalently crosslinked to the hydrogel. The authors used water-swellable inorganic clay NPs to increase the crosslinking density independently in PNIPAm hydrogels. The uniform distribution of the clay NPs within the flexible polymer network produced mechanically robust hydrogels. In 2004, Haraguchi et al. used an additional chemical crosslinker with clay nanofiller, which resulted in brittle hydrogels with lower mechanical strength. They found that the brittle properties were due to the inhomogeneity of the polymer chains introduced by chemical crosslinking [154]. In 2017, Creton et al. developed nano-engineered mechanically robust double crosslinked poly (N,N-dimethylacrylamide) (PDMA)-silica nanoparticle hydrogels with mild chemical crosslinking. These hydrogels have high compression strength [155]. In 2011, Gaharwar et al. also suggested similar kinds of results for nano-engineered mechanically robust prepared by using PEG diacrylate (PEG-DA)/silica nanoparticle-based chemically crosslinked hydrogels. They found that the chemical crosslinking leads to the form of an elastic network. The physical interaction between silica NPs and PEG-DA chains adds viscoelastic properties to the hydrogels. The elastic network and viscoelastic behaviour are responsible for the mechanically robust nature of prepared hydrogels [156]. In 2015, Xavier et al. prepared nano-engineered hydrogels using collagen and nanosilicates, a clay for bone tissue engineering. They found that with the addition of nanosilicates, the compressive modulus increased by 4 fold compared to collagen hydrogels [157].

In 2013, Campbell et al. developed injectable, in situ gelling composites by using pNIPAM−hydrazide functionalized superparamagnetic iron oxide NPs (SPIONs) to improve compressive modulus. Iron oxide NPs act as both fillers as well as crosslinkers to modify the mechanical properties of the composite with aldehyde–dextran. The developed composite hydrogel showed a high storage modulus of ~50 kPa with dynamic direct covalent crosslinking of SPIONs into a hydrogel network and non-cytotoxic in in vitro as well as in vivo [158]. An interesting injectable and flexible colloidal gel, with better mechanical properties in terms of elastic modulus, was prepared by Diba et al. in 2018 [159]. They developed an injectable colloidal hydrogel using gelatin NPs as reinforcement agents and building blocks. These gelatin NPs can easily accumulate within a hydrogel network and are reinforced with distinct poly-L-lactic acid (PLLA) fibres of different lengths. This reinforcement procedure forms an injectable and flexible gel with better mechanical properties. A 10-fold increase in elastic modulus was observed from 2.4 ± 0.2 to 23.1 ± 2.1 kPa in the resultant hydrogel, due to short-range hydrophobic interactions and ionic bonding in between the gelatin NPs. These bonds further interact with the carboxyl group of aminolyzed PLLA fibres and form a highly elastic colloidal gel.

In 2018, Boyer et al. developed silated hydroxypropylmethyl cellulose (Si-HPMC) based physically crosslinked hydrogels and showed effective reinforcement with nanosilicate fibres to enhance mechanical properties. The reinforcement with nanosilicate fibres (5 *w*/*v*%) enhanced the storage modulus of the hydrogels by 7 fold (~10 kPa), and compressive modulus by 4 fold (~24 kPa) as compared to hydrogels without nanosilicate fibres [160]. In 2014, Liu et al. developed four-arm PEG hydrogels by using nanosilicate fibres as a reinforcing agent and covalently crosslinked with dopamine through catechol oxidation. The reinforcement with 2 *w*/*v*% nanosilicate fibres increased the storage modulus by 1.5 fold (~15 kPa) [161]. In 2015, Casuso et al. studied the in situ fabrication of gold (Au) NPs within four-arm PEG–thiol hydrogels. They co-injected a mixture of PEG–thiol and excess gold chloride. The incorporation of 20 mol% gold NPs within a 20 wt% PEG–thiol hydrogel matrix resulted in a shear modulus of ~15 kPa, a ~22-fold increase as compared to 5 wt% PEG–thiol hydrogels prepared without any gold NPs [162]. In 2017, Fan et al. used antibacterial chitosan microspheres as reinforced agents to develop mechanically robust chemically crosslinked hydrogels of chondroitin sulfate/carboxymethyl. The hydrogels were crosslinked by using Schiff base chemistry. They reported that the incorporation of 2 *w*/*v*% of chitosan microspheres enhanced the compressive modulus by ~1.3 fold up to ~13 kPa [162].

**Table 2:** Storage modulus for mechanically reinforced injectable hydrogels.

S. No.	Hydrogel Components	Crosslinking Mechanism	Reinforcement	Storage Modulus	References
1.	QC, CCNC, β-GP	Physical	NPs	~1.3 kPa	[163]
2.	Peptide, GO	Physical	NPs	~1.7 kPa	[46]
3.	Chitosan-thiol, Dexamethasone	Covalent	DN	~2.2 kPa	[132]
4.	PEG, PNIPAM	Physical	Functionalization	~2.5 kPa	[164]
5.	POEGMA	Ionic–Covalent	DN	~3 kPa	[135]
6.	PNIPAM, Poly(vinylpyrrolidone)	Covalent	DN	~3 kPa	[165]
7.	POEGMA, Oligo(lactic acid)	Covalent	DN	~3.5 kPa	[166]
8.	Carboxymethyl cellulose, Cellulose nanocrystal	Chemical	NPs	~3.5 kPa	[167]
9.	Dexamethasone, Carboxymethyl cellulose, Cellulose nanocrystal	Physically and Chemically	NPs	~6.7 kPa	[168]
10.	Chitosan, Peptide microspheres	Chemical	NPs	~7.3 kPa	[169]
11.	Si-HPMC, Laponite	Physical	NPs	~10 kPa	[160]
12.	PEG-thiol, Gold	Physically and Chemically	NPs	~15 kPa	[162]
13.	PEG-3,4-Dihydroxyphenylalanine, Nanosilicates	Physically and Chemically	NPs	~15 kPa	[161]
14.	PEG, Cellulose, Alginate	Ionic	DN	~15 kPa	[134]
15.	Gelatin, PLA	Physical	NPs	~20 kPa	[159]
16.	Poly (vinyl) Alcohol, CPBA	Covalent	DN	~30 kPa	[91]
17.	POEGMA, Cellulose nanocrystal	Physically and Chemically	NPs	~40 kPa	[170]
18.	PNIPAM SPIONs, Dex	Chemical	NPs	~50 kPa	[158]
19.	QCS, PF127 micelles	Chemical	NPs	~53 kPa	[171]
20.	Peptide, PNIPAM	Physical	DN	~60 kPa	[129]

In 2021, Gupta et al. synthesized bioglass hydrogels by utilizing self-assembled peptide amphiphilic that act as a template for the deposition of bioactive glass. They reported that bundling of bioactive glass increases the stiffness of the native peptide fibres resulting in a 2-fold increase in elastic modulus. Hydrogels also possessed high yield stress (5780 Pa) indicating resistance to flow under applied stress in bioglass hydrogels and are favourable for load-bearing applications [172]. In 2021, Isik et al. developed a hybrid neuro-instructive hydrogel by combining a self-assembling peptide amphiphile (PA) and a photo-crosslinkable GelMA. Mechanically robust hydrogels were formed by combining electrostatic interaction and ultraviolet light crosslinking mechanisms. Results of dynamic oscillatory rheology and micromechanical testing showed that increasing the concentration of GelMA from 5 to 20% leads to an increase in stiffness from 8 kPa to 49 kPa making them suitable for load-bearing applications [163].

In 2022, Min et al. prepared thiol-conjugated chitosan–cysteine hydrogels crosslinked with amino-modified mesoporous bioglass nanoparticles. Authors reported that crosslinked hydrogels have much higher strength and elasticity in comparison to the hydrogels composed of single chitosan–cysteine and had an elastic modulus of around 8.4 kPa. They demonstrated that the higher strength and elasticity of the hydrogels were conducive to the synthesis of type-I collagen, showing potential for application in bone regeneration [164].

Overall, the approaches discussed above to improve the mechanical properties of hydrogels have certainly expanded the scope of hydrogel applications. However, not all hydrogels have shown feasibility for in vivo applications. Multistep synthesis routes by using toxic solvents remain impractical, and non-uniform degradation and swelling profiles with toxic byproducts, resulting in a drastic reduction of mechanical strength are unresolved tasks that still need to be addressed for designing nano-engineered mechanically robust injectable hydrogels for in vivo and clinical applications.

## 4. Mechanical Robust Hydrogels in Bone Tissue Regeneration

A wide range of injectable hydrogels, which can be delivered in a minimally invasive manner, are investigated for use in BTE. As explained above in Section 3.3, inorganic nanomaterials are usually introduced within hydrogels as reinforcement agents to improve mechanical properties and mineralization. In 2012, Fu et al. prepared an injectable thermoresponsive interconnected hydrogel by using PEG–PCL–PEG (PECE) copolymers, collagen and reinforced with nanohydroxyapatite (nHAp). They demonstrate through in vivo studies that the prepared hydrogel has good biocompatibility and exhibits better performance in guided bone regeneration than in the self-healing process, thus indicating its great promise for BTE [165]. In 2015, Dhivya et al. prepared an injectable thermoresponsive Zn-doped chitosan/nHA/β-glycerophosphate (βGP)-based hydrogel. Hydrogel without nHA was prepared to serve as the control. They reported increased protein adsorption, controlled swelling and degradation and osteoconductivity. These characteristics further promote the differentiation of mesenchymal stem cells (MSCs) into osteoblasts through upregulation of RUNX2 gene expression. They assessed the bone healing properties of the hydrogels in rat tibial defects. After 2 weeks, improved tissue organization was observed in tibiae treated with Zn-doped chitosan/nHAp/βGP incorporated hydrogels than in tibiae treated with Zn-doped chitosan/βGP hydrogels and control tibiae. They reported that the presence of nHAp in hydrogels leads to better wound closure and bone formation, as nHAp acts as a nucleating site for new bone formation [166].

In 2018, Thorpe et al. fabricated an injectable Laponite crosslinked poly-(N-isopropyl acrylamide) hydrogel reinforced with nHA that could effectively stimulate the osteogenic differentiation of human mesenchymal stem cells and osseointegration in a rat femoral defect [167]. In 2020, Lee et al. designed a GO-incorporated glycol chitosan (gC)-HA injectable hydrogel system via oxidation technique. Through oxidation, aldehyde groups were grafted onto the HA followed by vigorously mixing with gC. This will lead to the formation of gC/HA/GO hydrogel having a storage modulus ~9 kPa with a sol-to-gel transition time of less than one minute. The resultant hydrogels were implanted into a critical-sized rat calvarial defect model, and new bone formation extending inward from the margins of the defect after 4 weeks was confirmed by micro-CT and histological analysis [168].

In 2019, Fang et al. developed a strong (compressive strength ~6.5 MPa), biocompatible hydrogel by a simple one-step hydrophobic micellar copolymerization of acrylamide and crosslinked urethacrylate dextran (Dex-U). This was followed by the in situ mineralization of nHA over the hydrogel chains. They showed that the mineralized nHA layer promotes the adhesion and proliferation of osteoblasts, and effectively stimulates osteogenic differentiation in vitro. They found that in situ mineralized nHA on the hydrogel chains improved the mechanical properties of the hydrogels and promoted osteogenic differentiation of cells. They evaluated the hydrogels in vivo in a femoral condyle defect rabbit model and reported that a highly mineralized bone tissue was formed via direct bonding to the nHA mineralized PAAm/Dex-U hydrogel (nHA-PADH) interface. Mechanical properties analysis confirmed that the nHA-PADH hydrogel achieved a combination of superior mechanical properties and excellent osteointegration both in vivo and in vitro [169].

**Figure 6:**
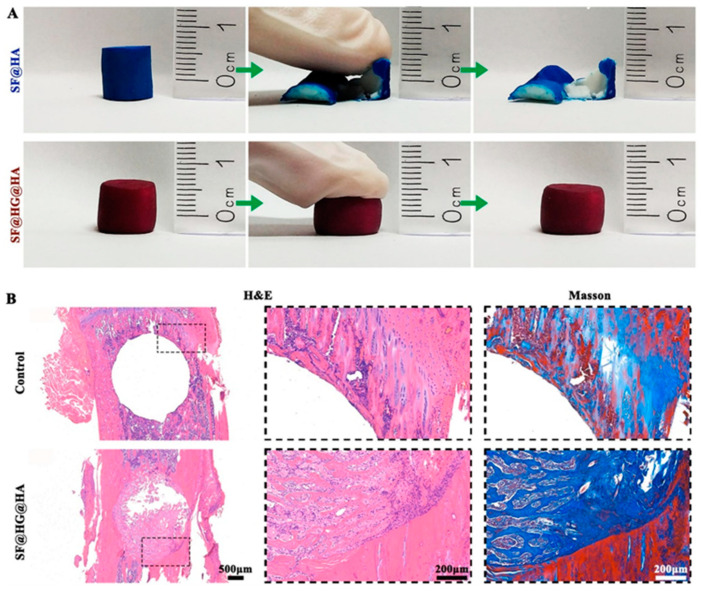
(**A**) Digital images of mechanical robust properties of SF@HG@HA during a compression test. (**B**) In vivo osteogenesis performance of SF@HG@HA. Histological analysis of newly formed bone with hematoxylin–eosin (H&E) and Masson’s trichrome (Masson) staining with their magnified view. Adapted with permission [130]. Copyright 2021 Elsevier.

In 2021, Bai et al. developed an inorganic/organic hybrid hydrogel using silk fibroin, a polymer template to tether β-cyclodextrin (host) and cholesterol (guest) monomers and labelled as SF@HG@HA. They reported that due to dynamic host–guest interactions, the prepared hydrogels are mechanically robust with self-healing potential when damaged, without the assistance of any external stimuli, mimicking the self-healing properties of native bone tissue (Figure 6A). Furthermore, the efficient energy dissipation mechanism provided by the host–guest crosslinking strategy endowed the hydrogel with robust mechanical properties to bear substantial mechanical loading. SF@HG@HA was shown to support human synovial fibroblast proliferation and osteogenic differentiation of rat MSCs in vitro and accelerate bone regeneration in critical-size rat femoral defects in vivo (Figure 6B). After 8 weeks, the degradation of SF@HG@HA enabled the ingrowth of cells and almost complete replacement of hydrogels with autologous tissue. The authors observed the layers of newly formed bone tissue inside the original defect area in the SF@HG@HA group, indicating better bone integration. Furthermore, the defect area was almost bridged by new bone tissue with mineralized collagen fibrils connecting the bone ends in the SF@HG@HA group [130].

In 2022, Yu et al. designed mechanically robust hydrogel with customized dual crosslinked networks using Polyhedral oligomeric silsesquioxane (POSS) surrounded by six disulfide-linked PEG shells and two 2-ureido-4[1H]-pyrimidinone (UPy) groups. Authors reported that multifunctional POSS units possessed favourable cytocompatibility and osteogenic properties that support cell attachment, spreading and proliferation. Periodontal ligament stem cells (PDLSCs) were used for biocompatibility studies and authors demonstrated that matrix stiffness is a key regulator for osteogenic differentiation. Mechanistically, they have shown that the key epigenetic regulator TET2 associated with HDAC1 for PDLSCs is upregulated on a stiff substrate and downregulated the E-cadherin transcription and WNT/β-catenin activation, and thus promotes the osteogenesis [170].

**Figure 7:**
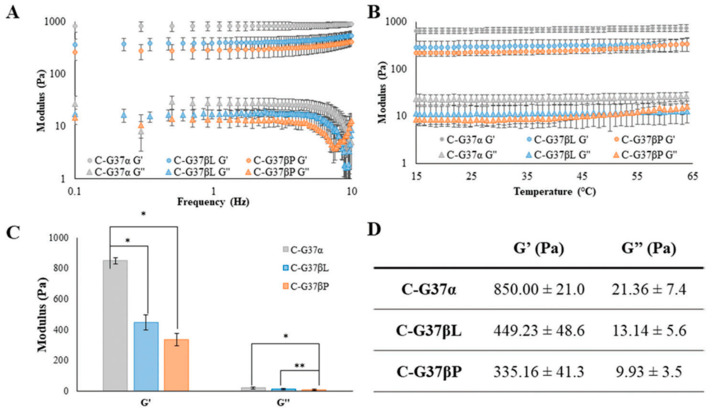
(**A**) Frequency sweep; (**B**) temperature sweep; (**C**) numerical values in graphical form; and (**D**) tabular form for storage and loss modulus for prepared hydrogels. Average ± STD, n = 3. * *p* < 0.01 and ** *p* < 0.05. Copyright 2023 Willey [171].

In 2023, Moore et al. developed a thermoresponsive, mechanically robust, biodegradable and biocompatible chitosan hydrogels crosslinked with genipin at 37 °C with different pre-injection crosslinking times. The hydrogels maintained a high percentage of swelling over 50 days before degrading in biologically relevant environments, demonstrating mechanical stability while remaining biodegradable. The authors characterized the prepared hydrogels against human keratinocyte cells. Long-term cell viability studies demonstrated that chitosan–genipin hydrogels have excellent biocompatibility (157 ± 11% cell viability) over 7 days, including during the hydrogel crosslinking phase. The authors demonstrated that pre-injection crosslinking does affect the swelling as well as mechanical properties with zero crosslinking time and has a higher storage modulus of 850 Pa (Figure 7). Authors highlight the use of these hydrogels for the use of these injectable, in situ crosslinking chitosan–genipin hydrogels as a proactive, biohybrid material with the potential for cell encapsulation [171].

Despite these favourable results, there is still a need to develop an injectable hydrogel that simulates the mechanical properties of the bone while maintaining proper accuracy in structural and biological properties. While many of the hydrogels developed to date have yet to progress into clinical trials, favourable preliminary in vitro and in vivo results indicate the use of these hydrogels for BTE applications with further optimization.

## 5. Conclusions and Future Directions

Mechanically robust injectable hydrogels are promising materials for bone tissue engineering and regeneration, owing to their minimal invasive properties and ability to match irregular bone defects. In this review article, we summarized novel injectable hydrogels prepared by a variety of fabrication techniques and materials (natural and synthetic). These hydrogels have certainly opened new frontiers in bone tissue engineering with the expansion of novel therapies and enabling innovative, interdisciplinary research that was previously limited by the weak mechanical properties of traditional treatments. Despite the tremendous advances in biomaterials for bone defects, many unmet challenges and needs continue to present obstacles to progress that are a more scientific and clinical translation of this approach. However, mechanically robustly reinforced with nanoparticles, injectable hydrogels are proven to be ready to use in clinics but still very much is at the initial stage. Body tissues are usually viscoelastic, whereas bone tissue engineering has widely focused on elastic materials with superior stiffness values and mechanical properties. The mechanical properties of hydrogels can be modified by reinforcing with nanomaterials. However, innovative chemical crosslinking also improves the mechanical potential by constructing an interpenetrating polymer network within hydrogel chains.

Moreover, non-uniform degradation and swelling profiles in media and buffers often impede mechanical strength and biological activity. As such, controlled degradation and swelling with non-toxic by-products are critical for achieving desirable therapeutic effects. Combining materials science with biology and chemistry will indeed lead to the formation of hydrogels with enhanced mechanical properties, bioactivity and refined micro/nanoarchitecture for robust use in bone regeneration. The cooperation between material researchers and professionals from chemistry and biomedical engineering will allow the enhancement of hydrogels with required physiochemical properties. The requirements of biomaterials for bone tissue engineering include similar biocompatibility with body tissue. Hydrogel selection is based on similarity to extracellular matrix, water content and minimal invasiveness of the technique used, and its potential to match irregular bone defects. Next-generation biomaterials are being rationally designed to fulfill the desired function by reacting actively to different stimuli. In the coming years, we can foresee the development of biomimetic hydrogels sustainable for longer periods with improved efficiency to restore patients with a high quality of life. With the advent of new methods and biomaterials, this area will continue to expand.

## Figures and Tables

**Table 1:** Commercially available minimally invasive bone graft materials.

Product Name	Brand	Type	Composition Components	Bioactive Properties	Reference Website
^β^Beta-bsm^®^	ZIMMER BIOMET (Wayne, IN, USA)	Injectable Paste	Noncrystalline calcium phosphate	Osteoconductive	https://www.zimmerbiomet.com/ (accessed on 29 September 2023).
Equivabone^®^		Moldable/Injectable Paste	DBM and calcium phosphate	Osteoconductive	https://www.zimmerbiomet.com/ (accessed on 29 September 2023).
nanOss^®^	Surgalign (Deerfield, IL, USA)	Mouldable/Injectable Putty	Nano-structured hydroxyapatite granules and an open-structured engineered collagen	Osteoconductive	https://surgalign.com (accessed on 29 September 2023).
Optecure^®^ +ccc	Exactech (Gainesville, FL, USA)	Injectable Paste	Demineralized Bone Matrix (DBM)	Osteoconductive	https://www.exac.com/ (accessed on 29 September 2023).
PRO-STIM^®^	WRIGHT Medical Group (Memphis, TN, USA)	Injectable inductive Paste	Calcium sulfate, calcium phosphate and DBM	Osteoconductive and osteoinductive	https://www.wright.com/ (accessed on 29 September 2023).
PRO-DENSE™		Injectable Paste	Calcium sulfate and calcium phosphate		https://www.wright.com/ (accessed on 29 September 2023).
ALLOMATRIX™		Mouldable/Injectable Putty	DBM, calcium sulfate hemihydrate and carboxymethylcellulose		https://www.wright.com/ (accessed on 29 September 2023).
Actifuse Flow	Baxter (Deerfield, IL, USA)	Implantable Solid/Paste	Silicate substituted calcium phosphate	Osteoconductive, osteostimulative, and provides accelerated bone growth	https://advancedsurgery.baxter.com/ (accessed on 29 September 2023).
CERAMENT^TM^	BoneSupport (Wellesley, MA, USA)	Mouldable/Injectable and Drillable Synthetic Bone Void Filler	40% hydroxyapatite, 60% calcium sulfate and the radio-contrast agent iohexol	Osteoconductive, promoting bone ingrowth	https://www.bonesupport.com/ (accessed on 29 September 2023).
Norian^®^SRS^®^	Synthes (Wayne, IN, USA)	Cement	Calcium phosphate	-	https://www.rch.org.au/ (accessed on 29 September 2023).
HydroSet™	Stryker (Kalamazoo, MI, USA)	Cement	Calcium phosphate	Osteoconductive	https://cmf.stryker.com/ (accessed on 29 September 2023).
Simplex^®^	Stryker (Kalamazoo, MI, USA)	Cement	PMMA	-	https://www.strykermeded.com/ (accessed on 29 September 2023).
SMARTSET^™^	DePuy Synthes (Wayne, IN, USA)			-	https://5.imimg.com/ (accessed on 29 September 2023).
PALACOS^®^	Heraeus (Hanau, Germany)			-	https://www.heraeus.com/ (accessed on 29 September 2023).

## Data Availability

No data were used for the research described in this article. The papers used are cited and given as references.
